# Supplementary material for: Diversity of Mitochondrial DNA Haplogroups and Their Association with Bovine Antral Follicle Count
Source: Animals (Basel). 2022 Sep 8;12(18):2350. doi: 10.3390/ani12182350 (PMC9495067; doi:10.3390/ani12182350)
Supplement: Supplementary file 1 [file animals-12-02350-s001.zip › Supplementary_Material.pdf]

Supplementary Materials

1 Supplementary Data

Supplementary data exclude the following figures and tables can be found in other files of this supplementary material.

2 Supplementary Figures and Tables

2.1 Supplementary Figures

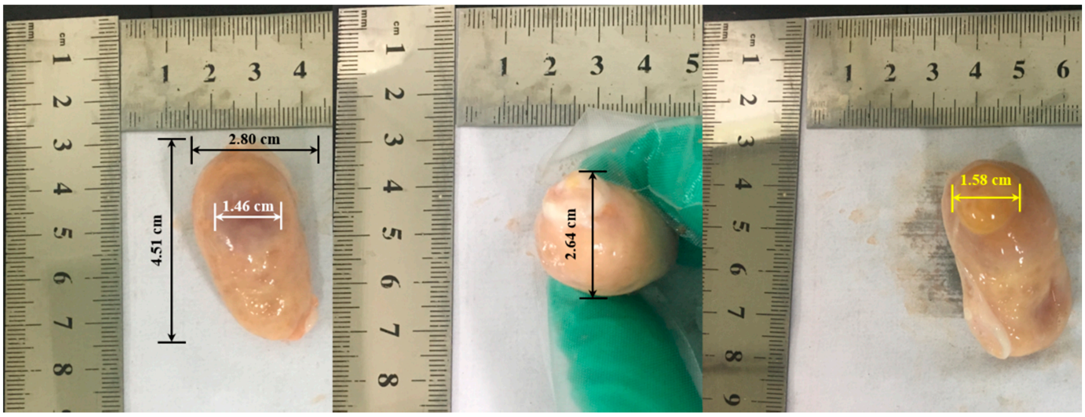

Figure S1. Morphological phenotypic traits of ovaries were measured by a double ruler. The black arrows and above records represent the length, width, height of ovaries, and the diameters of mature follicles and corpus luteum were shown in white and yellow arrows respectively.

2.2 Supplementary Tables

Table S1. The results of Shapiro-Wilk test for different haplogroups.

	Haplogroups	statistics	df	*P* values
**Antral follicle count (n = 115)**	**HG1 (n = 29)**	**0.650**	**29**	**4.29E-7**
	**HG2 (n = 86)**	**0.444**	**86**	**1.94E-16**
**Weight of ovaries (g) (n = 115)**	**HG1 (n = 29)**	**0.927**	**29**	**0.046**
	**HG2 (n = 86)**	**0.960**	**86**	**0.009**
Volume of ovaries (cm^3) (n = 115)	HG1 (n = 29)	0.956	29	0.264
	**HG2 (n = 86)**	**0.955**	**86**	**0.005**
Mean diameter of mature follicles (mm) (n = 115)	HG1 (n = 29)	0.978	29	0.776
	HG2 (n = 86)	0.985	86	0.418
**Number of corpus luteum (n = 115)**	**HG1 (n = 29)**	**0.681**	**29**	**1E-5**
	**HG2 (n = 86)**	**0.610**	**86**	**9.07E-14**
Mean diameter of corpus luteum (mm) (n = 115)	HG1 (n = 29)	0.930	29	0.056
	**HG2 (n = 86)**	**0.924**	**86**	**8.5E-4**

Table S2. The results of Levene's test for reproductive traits in two-way ANCOVA model.

	F	df1	df2	*P* values
**Antral follicle count (n = 115)**	**4.461**	**10**	**104**	**3.1E-4**
Weight of ovaries (g) (n = 115)	1.796	8	104	0.086*
Volume of ovaries (cm^3) (n = 115)	1.810	10	104	0.068
Mean diameter of mature follicles (mm) (n = 115)	0.761	10	104	0.665
**Number of corpus luteum (n = 115)**	**7.596**	**10**	**104**	**5.37E-9**
Mean diameter of corpus luteum (mm) (n = 115)	1.082	10	104	0.383

Note: *the *P* value of association of ovarian weight with different haplogroups was retrieved from two-way ANOVA without covariate: $Y_{ij} = \mu + H_i + S_j + e_{ij}$, where Y_{ij} is the overall mean value of ovarian weight, H_i is the fixed effect of haplogroup, S_j is the fixed effect of the types of CL, and e_{ij} is the random error.

Table S3. Interaction effects of different fixed factors with ovarian weight in two-way ANCOVA.

Interaction effects	Reproductive traits	df	Mean Square	F	*P* values
Haplotype * Ovarian weight	Volume of ovaries	1	15.472	.704	0.404
	Number of corpus luteum	1	.119	.196	0.659
	Mean diameter of corpus luteum	1	9.576	.338	0.562
	**Antral follicle count**	**1**	**3.140**	**14.398**	**2.56E-3**
	Mean diameter of mature follicles	1	38.210	2.286	0.134
CL types * Ovarian weight	Volume of ovaries	4	30.764	1.399	0.240
	Number of corpus luteum	4	.127	.210	0.932
	Mean diameter of corpus luteum	4	17.978	.635	0.639
	Antral follicle count	4	.472	2.165	0.079
	**Mean diameter of mature follicles**	**4**	**62.776**	**3.755**	**0.007**
Haplotype * CL types * Ovarian weight	Volume of ovaries	4	14.910	.678	0.609
	Number of corpus luteum	4	.006	.010	1.000
	Mean diameter of corpus luteum	4	23.389	.826	0.512
	Antral follicle count	4	.104	.477	0.752
	Mean diameter of mature follicles	4	19.417	1.161	0.333

Table S4. Parameters estimation of dependent variables in two-way ANCOVA.

Dependent variables	Parameters	B	SE	t	*P* values	95% Confidence level	
						Lower Bound	Upper Bound
Volume of ovaries	Intercept	-0.534	5.891	-0.091	0.928	-12.224	11.156
	Ovarian weight	1.457	0.356	4.098	0.000	0.751	2.163
Number of corpus luteum	Intercept	1.179	0.978	1.206	0.231	-0.761	3.119
Mean diameter of mature follicles	Intercept	-7.064	6.686	-1.057	0.293	-20.331	6.204
	[CL types=3]	16.185	7.114	2.275	0.025	2.068	30.303
	Ovarian weight	1.003	0.404	2.486	0.015	0.203	1.804
Antral follicle count	Intercept	1.572	0.587	2.680	0.009	0.408	2.737
	[CL types =3] * Ovarian weight	0.089	0.038	2.366	0.020	0.014	0.164
Mean diameter of mature follicles	Intercept	-1.832	5.137	-0.357	0.722	-12.026	8.363
	[CL types =2]	21.357	6.531	3.270	0.001	8.396	34.317
	Ovarian weight	1.180	0.310	3.805	0.000	0.564	1.795
	[CL types =0] * Ovarian weight	-0.806	0.325	-2.482	0.015	-1.451	-0.162
	[CL types =2] * Ovarian weight	-1.420	0.394	-3.604	0.000	-2.202	-0.638
	[CL types =3] * Ovarian weight	-0.889	0.329	-2.702	0.008	-1.542	-0.236

Table S5. Association of haplogroups with morphological phenotypic traits of ovaries.

	HG1	HG2	*P* values
Weight of ovaries (g)<br>(n = 115)	13.71±1.23<br>(n = 29)	13.17±0.55<br>(n = 86)	0.268*
Volume of ovaries (cm^3)<br>(n = 115)	19.25±1.92<br>(n = 29)	18.77±0.99<br>(n = 86)	0.887
Mean diameter of mature follicles (mm)<br>(n = 115)	11.94±0.81<br>(n = 29)	13.26±0.49<br>(n = 86)	0.446
Number of corpus luteum<br>(n = 115)	1.62±0.18<br>(n = 29)	1.62±0.12<br>(n = 86)	0.768
Mean diameter of corpus luteum (mm)<br>(n = 115)	11.90±1.44<br>(n = 29)	10.71±0.83<br>(n = 86)	0.617

Note: *the *P* value of association of ovarian weight with different haplogroups was retrieved from Scheirer–Ray–Hare test without covariate: $Y_{ij} = \mu + H_i + S_j + e_{ij}$, where Y_{ij} is the overall mean value of ovarian weight, H_i is the fixed effect of haplogroup, S_j is the fixed effect of the types of CL, and e_{ij} is the random error.

Table S6. Results of the Scheirer-Ray-Hare test of between-subjects effects on antral follicle count.

**Sources**	***df***	**Sum Sq**	**H**	***P* values**
**Haplogroup**	**1**	**3337**	**5.9960**	**0.01434**
Types of CL	5	2320	4.1683	0.52545
Haplogroup * Types of CL	4	1427	2.5643	0.63315
Error	104	56335		
Total	114			
